# Blast Transformation in Myeloproliferative Neoplasms: Risk Factors, Biological Findings, and Targeted Therapeutic Options

**DOI:** 10.3390/ijms20081839

**Published:** 2019-04-13

**Authors:** Alessandra Iurlo, Daniele Cattaneo, Umberto Gianelli

**Affiliations:** 1 Hematology Division, Foundation IRCCS Ca’ Granda Ospedale Maggiore Policlinico, and University of Milan, 20122 Milan, Italy; cattaneo.daniele@alice.it; 2 Division of Pathology, Department of Pathophysiology and Transplantation, Foundation IRCCS Ca’ Granda Ospedale Maggiore Policlinico, and University of Milan, 20122 Milan, Italy; umberto.gianelli@unimi.it

**Keywords:** myeloproliferative neoplasms, blast phase, secondary acute leukemia, mutations, targeted therapies

## Abstract

Myeloproliferative neoplasms represent a heterogenous group of disorders of the hematopoietic stem cell, with an intrinsic risk of evolution into acute myeloid leukemia. The frequency of leukemic evolution varies according to myeloproliferative neoplasms subtype. It is highest in primary myelofibrosis, where it is estimated to be approximately 10–20% at 10 years, following by polycythemia vera, with a risk of 2.3% at 10 years and 7.9% at 20 years. In essential thrombocythemia, however, transformation to acute myeloid leukemia is considered relatively uncommon. Different factors are associated with leukemic evolution in myeloproliferative neoplasms, but generally include advanced age, leukocytosis, exposure to myelosuppressive therapy, cytogenetic abnormalities, as well as increased number of mutations in genes associated with myeloid neoplasms. The prognosis of these patients is dismal, with a medium overall survival ranging from 2.6–7.0 months. Currently, there is no standard of care for managing the blast phase of these diseases, and no treatment to date has consistently led to prolonged survival and/or hematological remission apart from an allogeneic stem cell transplant. Nevertheless, new targeted agents are currently under development. In this review, we present the current evidence regarding risk factors, molecular characterization, and treatment options for this critical subset of myeloproliferative neoplasms patients.

## 1. Introduction

The *BCR-ABL1*-negative myeloproliferative neoplasms (MPNs) are clonal disorders of the hematopoietic stem cell, mainly characterized by hyperproliferative bone marrow with varying degrees of reticulin/collagen fibrosis, extramedullary hematopoiesis, abnormal peripheral blood count, and constitutional symptoms. They include polycythemia vera (PV), essential thrombocythemia (ET), and primary myelofibrosis (PMF) [1].

The major causes of morbidity and mortality in these patients are most commonly represented by thrombo-hemorrhagic events and less frequently by infectious complications, and/or transformation to blast phase, often termed secondary acute myeloid leukemia (AML) or blast-phase MPN (MPN-BP) [2,3]. The term MPN-BP has been proposed by the International Working Group for Myelofibrosis Research and Treatment (IWG-MRT) to reflect the occurrence of leukemic transformation in the classical *BCR-ABL1*-negative MPNs. This setting now represents the principal clinical challenge in these diseases.

The frequency of leukemic evolution varies according to MPN subtype. It is highest in PMF, where it is estimated to be approximately 10–20% at 10 years, following by PV, with a risk of 2.3% at 10 years and 7.9% at 20 years [4,5,6]. On the contrary, transformation to AML is relatively uncommon in ET. In detail, considering different studies, the incidence of AML evolution in ET has varied widely, from less than 1% to almost 10%. More precisely, the 10-year rates from earlier studies have ranged from 2.6% [7] to 8.3–9.7% [8,9,10]. However, after the clear definition of prefibrotic PMF and its precise distinction from ET, a remarkably lower rate of leukemic evolution of less than 1% at 10 years and 2% at 15 years in WHO-defined ET has been defined [11,12]. In the literature, no significant difference in leukemic evolution has instead been reported between primary and secondary MF. Furthermore, we must consider that both PV and ET can also directly evolve into AML without going through an intermediate fibrotic stage. These data are also supported by an important multicenter study with more than 1500 *BCR-ABL1*-negative MPN patients, where the cumulative incidence of MPN-BP was 0.038 for ET, 0.068 for PV, and 0.142 for PMF [2].

The prognosis of these patients is dismal, with a medium overall survival (OS) ranging from 2.6–7.0 months [13]. Currently, there is no standard of care in the management of MPN-BP and no treatment can significantly prolonged survival and/or obtain a hematological remission apart from an allogeneic stem cell transplant (ASCT).

In this review, we present the current evidence regarding molecular characterization and treatment options for this subset of MPN patients.

## 2. Clinical Risk Factors

Even though risk factors for leukemic evolution in *BCR-ABL1*-negative MPNs vary according to the specific MPN subtype, they generally include advanced age, leukocytosis, exposure to myelosuppressive therapy, cytogenetic abnormalities, as well as an increased number of mutations in genes associated with myeloid neoplasms.

In particular, independent risk factors for leukemic transformation in PMF included peripheral blood (PB) blast >3% and platelet count <100 × 10^9^ L [14]. Using these risk factors, leukemic transformation was reported in only 6% of the patients if both risk factors were absent and in 18% of the patients if one or both risk factors were present. Leukocytosis (>30 × 10^9^ L), and red blood cell (RBC) transfusion need were also associated with an increased risk of leukemic transformation in PMF, with an incidence at 7.4 × 100 persons per year in RBC-transfused patients vs. 1.5 × 100 persons per year in non-transfused patients (*p* < 0.001) [15,16]. Treatments with hydroxyurea, thalidomide, or many other drugs were not found to be associated with an increased risk of leukemic transformation, even though a potential detrimental effect from erythropoiesis stimulating agents and danazol was reported. Other proposed risk factors include increased serum interleukin 8 [17], or C-reactive protein levels, age >65 years, and PB blast count >1% [18].

Unfavorable karyotype together with thrombocytopenia were then identified as being the most important risk factors for leukemic evolution in PMF [19]. The latter was reported in 6% and 12% of patients at 5 and 10 years, respectively, in the absence of any risk factor, whereas it was substantially higher in patients with one or more risk factors, i.e. 18% and 31% at 5 and 10 years, respectively [15]. More recent studies have confirmed the adverse effect of specific cytogenetic abnormalities, with a 2-year rate of leukemic transformation of 29.4% in patients with a monosomal karyotype as compared with 8.3% if a complex karyotype was documented [20].

With regards to PV, historical treatments, such as P32, chlorambucil, or pipobroman, have been clearly demonstrated to be associated with a higher risk of leukemic transformation [21,22]. Other factors, including age >61 years [23,24], leukocyte count >15 × 10^9^ L [23,25], and an abnormal karyotype [23] have also been associated with a higher risk of leukemic transformation. In contrast, there was no objective evidence in recent studies that hydroxyurea is leukemogenic [21,23], despite the controversy surrounding this agent and the issue of leukemogenicity.

Concerning ET, Gangat et al. [26] identified anemia, extreme thrombocytosis (>1000 × 10^9^ L), and age as independent risk factors for leukemic transformation in this subset of MPN patients. In detail, the risk of leukemic transformation was low at 0.4% if both the aforementioned risk factors were absent, and was significantly higher at 4.8% and 6.5% in the presence of one or both risk factors, respectively (*p* < 0.001). Interestingly, many important retrospective case series have supported the absence of any convincing evidence for drug leukemogenicity in ET [27], even though reports to the contrary have also to be mentioned [8].

## 3. Biological Risk Factors

As reported above, a complex/monosomal karyotype represents an important risk factor for leukemic evolution, as a favorable karyotype is infrequent in MPN-BP.

Concerning the molecular profile, if driver mutations are important in MPN pathogenesis, they also have a critical prognostic role in terms of leukemic transformation. It is best recognized for PMF patients, where a higher risk has been associated with the so-called triple-negative molecular status (i.e., with no *JAK2*, *CALR*, or *MPL* mutations) [2].

However, it is now clear that *BCR-ABL1*-negative MPNs are molecularly complex and are associated with several other recurrent gene mutations, including those involving epigenetic modifiers and spliceosome machinery (Table 1). Using a candidate gene approach, five mutated genes including *ASXL1*, *SRSF2*, *EZH2*, *IDH1*, and *IDH2*, which are reported to occur in 25–30% of all PMF patients, were associated with shorter OS and leukemia-free survival (LFS), defining a high-molecular risk (HMR) category [28]. In detail, the presence of two or more HMR mutations was associated with the worst outcome, in particular with a significantly shortened LFS (HR 6.2, 95% CI 3.5–10.7) [2]. The contribution of these mutations in conferring high risk for leukemic transformation was reported in other studies as well [28,29,30,31,32]. Subsequently, a Mayo Clinic study of targeted sequencing in PMF identified mutations in other genes, such as *CBL*, *RUNX1*, *CEBPA*, *SH2B3*, and *KIT*, as interindependent risk factors for OS and LFS [30]. With regards to PV, *ASXL1*, *SRSF2*, *RUNX1*, and *IDH2*, these were identified as adverse variants or mutations based on their effect on OS, LFS, or MF-free survival [33]. More recent studies have examined their role also in the development of leukemia in ET, identifying *TP53*, *EZH2*, *SRSF2*, and *IDH2* variants or mutations as being associated with a higher risk of leukemic transformation [33].

## 4. Morphological and Histological Characteristics of MPN Progression

**Figure 1:**
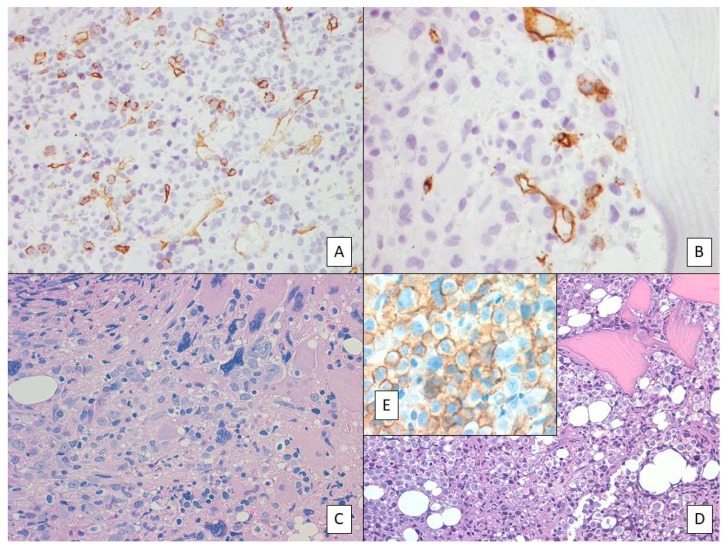
(**A**) Primary myelofibrosis in accelerated phase (AP). Myeloid hyperplasia with increased number of immature precursors and blasts together with large to giant megakaryocytes with hyperlobulated nuclei. (**B**). CD34 immunostaining highlighting the increased number of blasts and their cluster formation. (**C**) Paratrabecular localization of CD34-positive blasts suggests myeloproliferative neoplasm (MPN)-AP. (**D**) AML (M6-FAB) evolution of a case of polycythemia vera (PV). (**E**) Anti-E-cadherin immunostaining documenting the protein expression in the majority of acute myeloid leukemia (AML) (FAB-M6) blasts.

In the updated version of the WHO classification of tumors of the hematopoietic and lymphoid tissues [1], specific criteria to define the accelerated phase (AP) and BP of *BCR-ABL1*-negative MPNs have been included. Accordingly, the finding of 10–19% of blasts in the PB and/or in the bone marrow (BM), as well as the immunohistochemical detection of an increased number of CD34+ cells with cluster formation and/or an abnormal endosteal location in the BM [34,35], indicate an AP of the disease (Figure 1A). This definition clearly highlights the importance of a proper evaluation of both BM aspirate and trephine biopsy. In the latter, a very detailed evaluation of the CD34+ blasts needs to be performed, not only limited to the simple assessment of their percentage, but also of their clustering and abnormal localization near the bony trabeculae (Figure 1B,C). Due to their clinical importance, either blasts clustering or their paratrabecular localization are two concepts that need to be “metabolized” by both the pathologists and hematologists. Finally, the detection of more than 20% of blasts is diagnostic of BP. However, discordance in the content of PB vs BM is often seen.

Blast-phase MPNs commonly involved the myeloid lineage, being the lymphoid lineage only rarely involved. The morphological and cytogenetic characteristics of MPN-BP have been reported to be different from primary (de novo) AML. According to the French-American-British (FAB) classification of AML, erythroleukemia (FAB-M6) (Figure 1D,E) and megakaryoblastic leukemia (FAB-M7) were the most common subtype reported in MPB-BP. In this context, it must be remembered that even if rarely, patients with MPN may present at diagnosis in the AP or BP of the disease [1,36].

Interestingly, the percentage of blasts suggesting MPN progression is going to be investigated in a cooperative European and American effort, also involving our group. In detail, 114 patients with a diagnosis of *BCR-ABL1*-negative MPN have been collected. Inclusion criteria included: increased PB (≥2%) and/or BM (≥5%) blasts, presence of dysplastic features, persistent leukocytosis (≥15 × 10^9^ L) or monocytosis (≥1 × 10^9^ L), and extreme thrombocytosis (≥1000 × 10^9^ L). On follow-up, 22 (22%) patients developed AP and 19 (19%) BP. Forty-seven patients (41%) expired after a median follow-up of 11 months from disease progression, as compared to 2/40 (5%) control patients (*p* < 0.0001). Furthermore, there was no significant difference in OS between patients with AP and other types of progression. Accordingly, a review of the blasts threshold to define AP of *BCR-ABL1*-negative MPNs could be proposed [37].

However, types of progression other than blast percentage increase have been described in MPNs. In particular, a cohort of 10 PMF patients developed absolute monocytosis during the disease course. It arose at a median interval of 42 months from diagnosis (range: 1-180), persisting for a median period of 23 months (range: 2–57). Among these patients, five died after developing monocytosis (range: 20–188 months), and two experienced worsening disease with transfusion dependence. Interestingly, four of nine patients analyzed showed *KRAS* mutation in codon 12 or 13 with a low allelic burden. On this basis, the development of monocytosis during PMF has been proposed as an AP of the disease [38]. Clearly, a previous diagnosis of chronic myelomonocytic leukemia as a de novo disease should be ruled out. The latter is a myelodysplastic/myeloproliferative neoplasm of variable, but usually unfavorable, prognosis which is mainly characterized by the presence of absolute monocytosis (≥1 × 10^9^ L), sustained for more than 3 months, together with dysplastic features involving one or more myeloid lineages [1].

Considering instead PV patients, in a previous study involving the same cooperative group, absolute neutrophilic leukocytosis (≥13 × 10^9^ L) developed at or around the time of evolution in post-polycythemic MF, was associated with a worse outcome: four patients out of 10 died after developing leukocytosis and one experienced worsening disease. In addition, when compared with a control group of post-polycythemic MF patients (*n* = 23) who never developed persistent leukocytosis, the former showed a shorter OS, suggesting that persistent leukocytosis could be associated with an overall more aggressive course of the disease [39]. Interestingly, the development of leukocytosis was not associated with changes in *JAK2* and *BCR-ABL1* status or cytogenetic evolution. Furthermore, the mutational status of *CSF3R*, *SETBP1*, and *SRSF2*, genes associated with other chronic myeloid neoplasms with neutrophilic leukocytosis, was investigated, but no mutation was detected.

## 5. Karyotype

Karyotype has an important role in prognosis, it having an adverse effect. Usually, it is reported as abnormal and most often is labeled as “high risk”, based on monosomal karyotype or monosomy 7, single or multiple abnormalities including inv(3)(q21.3q26.2)/t(3;3)(q21.3;q26.2) or i(17)(q10). In addition, the cytogenetic profile was similar between post-PMF and post-PV/ET MPN-BP [40].

## 6. Molecular Profile

According to what has been previously reported, analysis of paired samples in chronic phase MPN vs. MPN-BP has clearly demonstrated that more than one signaling pathway is associated with leukemic transformation. In addition, *JAK2*-mutated chronic phase disease transformed into *JAK2*-mutated MPN-BP in some patients, whereas in other cases the *JAK2* mutation was not detected further [41,42]. Accordingly, the transforming event which leads to AML could occur in a pre-*JAK2*-mutated ancestral clone, or chronic phase MPN could be biclonal from its outset.

The mutational profile of MPN-BP is different from that of de novo AML. Indeed, in contrast to the latter, in which mutations in *FLT3*, *NPM1*, and *DNMT3A* are predominate, MPN-BP is frequently associated with mutations in *IDH1*, *IDH2*, *TET2*, *SRSF2*, *ASXL1*, and *TP53* [43,44,45,46]. Knowledge of the molecular events and clonal dynamics associated with leukemic transformation in MPNs has been greatly improved in recent years by high-throughput sequencing techniques. In particular, in a recent study which analyzed serial samples from 143 MPN patients by means of next generation sequencing (NGS), it was demonstrated that most mutations were already present at MPN diagnosis, with only very few additional mutations being acquired during the follow-up. Of note, in some patients who evolved to the BP of their disease, *TP53* somatic mutations were present for many years at a low allelic burden in the chronic phase of the disease, with loss of heterozygosity resulting in clone expansion and AML transformation [47].

## 7. Therapy

*BCR-ABL1*–negative MPNs in accelerated or blast phase of the disease have been associated with a poor response to therapy and severely shortened survival [19,48,49].

Conventional antileukemic therapy has limited efficacy in this setting for patients, and current therapeutic strategies for MPN-BP and AP rarely offer more than palliative benefit [6,49,50,51]. Thus, MPN-BP represents an area of urgent clinical need.

At present, ASCT is the best therapeutic option, but initially requires intensive chemotherapy to reduce the disease burden to become eligible. However, in most patients, ASCT is not feasible, mainly due to advanced age, significant comorbidities, and poor performance status, and consequently fewer than 10% of patients undergo a transplant [6].

Patients ineligible for ASCT are treated with supportive therapy and non-intensive chemotherapy, like hypomethylating agents (Table 2), because the benefit of intensive therapy is now limited only to patients who can undergo a transplant.

## 8. Supportive Therapy

This approach included antibiotics, RBC, and/or platelet transfusions, and oral chemotherapy with hydroxyurea for prevention of leukostasis. However, none of these regimens has been shown to be able to produce an effective response, and the median OS with these approaches is only 2.0 months (range 0.0–20.1 months) [59].

## 9. Hypomethylating Agents

Effectively-targeted chemotherapeutic agents that are currently available include two hypomethylating agents that have been approved by the US FDA for MPN-BP. These are 5-azacytidine (azacytidine) and 5-aza-2′deoxycitidine (decitabine). The rationale for their use is based on the observation that hypermethylation of p15/p16 gene promoter sites has been reported in patients with MPN-BP, but not in the chronic phase of these diseases [60]. These genes block normal myeloid cells differentiation, thus, their inhibitors are used for the treatment of these diseases. However, their exact mechanism of action has yet to be understood. Both agents incorporate into DNA, with azacytidine additionally incorporating into RNA. Then, they form a covalent complex with the DNA methyltransferase enzyme, leading to its trapping and degrading, and to the subsequent DNA hypomethylation. Even though at very high doses the cytotoxic effects of these agents predominate, lower doses allow hypomethylation, resulting therefore in epigenetic modulation [61].

### 9.1. Azacitidine

Azacitidine is administrated at a dosage of 75 mg/m^2^ subcutaneously for 7 days every 28 days, for up to 6 cycles, consecutively.

In a study of 26 azacitidine-treated patients, a 38% response rate (median time to response: 9 months) with an OS of 8 months was reported. Interestingly, the authors observed better responses in post-ET, as opposed to post-PV, MPN-BP, though with no significant OS difference [52]. Another study which reviewed MPN-BP patients treated with azacitidine found a median OS better than that of their historical controls (9.9 months). Once again, the median survival of patients achieving a complete response (CR) was even better at 19.6 months [53].

Therefore, it can be concluded that hypomethylating agents should be considered for patients who are ineligible for ASCT [54]. In addition, potential roles of this agent include providing disease control until an ASCT donor is available, acting as adjuvant for induction therapy (in particular for patients with a complex karyotype), and serving as a substitute agent in intensive chemotherapy during the pre-ASCT period [62].

### 9.2. Decitabine

The use of decitabine has demonstrated efficacy with a median survival beyond 9 months [63]. It is administrated at a dosage of 20 mg/m^2^ intravenously over 1 h for 5 days every 28 days, for up to 6 cycles, consecutively. This hypomethylating agent has been used also in MF to alleviate splenomegaly and anemia [59,60].

Decitabine compares favorably with either supportive care or intensive induction chemotherapy. However, it requires support with both RBC and platelet transfusions, owing to the common adverse effect of myelosuppression.

Combination therapy of decitabine and the *JAK2* inhibitor ruxolitinib has also shown promising activity [55,56]. In particular, in a recent report, the overall response rate by protocol-defined criteria (complete remission with incomplete count recovery + partial remission) was 53% [57]. This association was in general well tolerated and demonstrated potentially promising clinical activity [58].

## 10. Other Therapies

Despite these encouraging findings, the response duration of hypomethylating agents is short, and therefore, other therapies need to be evaluated as well.

Among the most promising ones, CPX-351 is a liposome formulation of cytarabine and daunorubicin which are encapsulated in a fixed 5:1 molar ratio. This delivery system improved drug concentration in BM and its uptake into blasts, determining superior antileukemic efficacy in vivo [64,65]. In addition, this new formulation seems to be able to overcome other resistance mechanisms, such as P-glycoprotein efflux and other first-pass metabolism [66]. In May 2016, it received US FDA approval for therapy-related or secondary AML. Moreover, the improved survival (median, 10 months with CPX-351 vs 6 months with the standard “7 + 3” therapy; *p* = 0.005) reported in the relevant phase III trial of CPX-351 involved older patients and high-risk AML, including therapy-related AML, and with antecedent myelodysplastic syndrome (MDS) or chronic myelomonocytic leukemia [67]. However, frail subjects may be particularly prone to mortality, mainly due to sepsis, hemorrhagic complications, and myelosuppression. As the practicality of dose adjustment is now limited by the availability of a fixed molar ratio, the most reasonable strategy would be to use full-dose CPX-351 only in relatively fit patients who are expected to tolerate it [68].

Another possible therapeutic target is now represented by the family of bromodomain and extraterminal domain (BET) proteins. They are chromatin reader proteins that contain N-terminal, double-tandem bromodomains that bind to the acetylated lysine on the nucleosomal histones and transcription factors [69]. Their inhibitors (BETis) target epigenetic proteins in cancer and were studied in patient-derived MPN blast progenitor cells and exhibited activity-inducing apoptosis and inhibiting growth. In detail, the authors demonstrated that co-treatment with BETi and *JAK* inhibitors is synergistically lethal against AML cells sensitive to *JAK* inhibitors, whereas combined therapy with BETi and heat shock protein 90 inhibitors exerts synergistic lethality against AML cells which are resistant to *JAK* inhibitors [70].

Other agents which are currently under investigation include histone deacetylase inhibitors, target therapy against *IDH2* (enasidenib), CD33 (gemtuzumab ozogamycin), or BCL-2 (venetoclax) [71].

Histone deacetylase inhibitors, including panobinostat, have been evaluated in small studies of MPN patients, showing an initial clinical improvement and a good safety profile [72,73].

Enasidenib effectively suppressed 2-hydroxyglutarate production, thus releasing myeloid blasts from differentiation block [74]. As an orally administered drug, it produced complete response (CR) and complete remission with incomplete hematologic recovery (CRi) in 26.6% of patients, with a distinct toxicity profile, the most important being *IDH* inhibitor-associated differentiation syndrome [75].

Gemtuzumab ozogamycin initially received FDA approval for relapsed AML in May 2000, but was soon retired due to a lack of benefit when added to standard therapy in a phase III confirmatory study [76]. It was then reapproved in September 2017, based on new findings of improved event-free survival and 5-year OS and a reasonable safety profile [77,78].

The BCL-2 inhibitor venetoclax was only partially effective as monotherapy in relapsed/refractory AML (19% CR/CRi) [79]. Nevertheless, when used in combination with hypomethylating agents, CR/CRi rate increases up to 62% [80,81]. Importantly, responses were achieved rapidly, and early mortality was low.

## 11. Conclusions

Leukemic evolution represents a critical complication in the natural history of *BCR-ABL1*-negative MPNs, with a frequency varying according to the specific MPN subtype. It is highest in PMF, following by PV and finally by ET.

Many different risk factors for leukemic evolution has been recognized, among them, a prominent role must be attributed to biological findings. In particular, a complex/monosomal karyotype represents an important risk factor, as a favorable karyotype is infrequent in MPN-BP. In addition, several recurrent gene mutations, including those involving epigenetic modifiers and spliceosome machinery, are involved in this phase of the disease.

The conventional antileukemic therapy has limited efficacy in this setting of patients and current therapeutic strategies rarely offer more than a palliative benefit. Nevertheless, based on new molecular acquisitions, new targeted agents are currently under development. In this context, participation of these patients in clinical trials should be strongly encouraged.

## Figures and Tables

**Table 1:** Biological Risk Factors.

Gene	Gene Function	Chromosome Location	Prognostic Significance	References
*ASXL1*	Epigenetic regulation	20q11.1	Adverse in PV and PMF	[28,29,30]
*SRSF2*	mRNA processing	17q25.1	Adverse in PV, ET and PMF	[28,30]
*EZH2*	Epigenetic regulation	7q36.1	Adverse in ET and PMF	[28,30]
*IDH1*	Epigenetic regulation	2q33.3	Adverse in PMF	[28,30,31,32]
*IDH2*	Epigenetic regulation	15q26.1	Adverse in PV, ET and PMF	[28,30,31,32]
*CBL*	Cell signaling pathways	11q23.3	Adverse in PMF	[30]
*RUNX1*	Transcriptional regulation	21q22.12	Adverse in PV and PMF	[30]
*CEBPA*	Transcriptional regulation	19q13.1	Adverse in PMF	[30]
*SH2B3*	mRNA processing	12q24	Adverse in ET and PMF	[30]
*KIT*	Tyrosine kinase receptor	4q11	Adverse in PMF	[30]
*TP53*	Transcriptional regulation	17p13.1	Adverse in ET	[33]

**Table 2:** Conventional therapeutic options.

Treatment Approach	Patient Population	Results	Survival	References
HMAs	MPN-BP (*n* = 26); MPN-AP (*n* = 28)	ORR, 52% with azacitidine	11 months	[52]
	MPN-BP (*n* = 19)	ORR, 47% with azacitidine	9.9 months	[53]
	MPN-BP (*n* = 21); MPN-AP (*n* = 13); PMF (*n* = 11)	ORR in MPN-BP, 29% with decitabine	6.9 months	[54]
JAK inhibition	R/R AML (*n* = 38), including MPN-BP (*n* = 18)	CR/CRi 17%	NR	[55]
	R/R AML (*n* = 28), including MPN-BP (*n* = 7)	ORR, 0%	NR	[56]
JAK inhibition + HMAs	MPN-BP/AP (*n* = 21)	ORR; 33%	10.4 months	[57]
	MPN-BP (*n* = 10)	ongoing	NR	[58]

Abbreviations: HMAs, hypomethylating agents; ORR, overall response rate.
